# Evaluation of Magnetic Ureteric Stents: A Systematic Review and Meta-Analysis

**DOI:** 10.7759/cureus.75126

**Published:** 2024-12-04

**Authors:** Peter Estaphanous, Ahmed Elbassyiouny, Youstina Makar

**Affiliations:** 1 Urology, University Hospitals Coventry and Warwickshire, NHS Trust, Coventry, GBR; 2 Medicine and Surgery, University Hospitals Coventry and Warwickshire, NHS Trust, Coventry, GBR

**Keywords:** magnetic-tip ureteric stents, magnetic ureteric stents, minimally invasive surgical procedures, ureteric stent, ureteric stent removal

## Abstract

Magnetic ureteric stents offer a novel approach for simplifying stent removal, minimizing patient discomfort, and reducing procedural burdens. This systematic review and meta-analysis synthesized evidence from 12 studies involving 1,297 patients to evaluate the efficacy and safety of magnetic stent removal compared to conventional methods. Key outcomes included reductions in procedural pain scores, shorter removal times, and high patient satisfaction. Complication rates for magnetic stents were comparable to conventional stents, while cost analysis favored magnetic removal due to reduced resource utilization. These findings suggest magnetic stents are a safe, effective, and patient-friendly alternative. Further research with larger sample sizes is warranted to confirm these benefits.

## Introduction and background

Ureteric stents are commonly employed to ensure adequate urinary drainage following procedures such as ureteroscopy or address ureteral obstruction caused by conditions such as kidney stones, tumors, or fibrosis [1]. They help maintain urine flow from the kidneys to the bladder and prevent complications like hydronephrosis and infection [2]. However, conventional stent removal typically requires cystoscopy, an endoscopic procedure that is often uncomfortable for patients, resource-intensive for healthcare providers, and associated with high procedural costs [3]. These challenges have led to the development of magnetic ureteric stents, which aim to simplify the removal process, reduce discomfort, and enhance the overall patient experience by allowing less invasive options for retrieval [4].

Magnetic ureteric stents are designed with a magnetic tip that allows for non-invasive retrieval using a handheld magnetic device [5]. This design innovation eliminates the need for cystoscopy in many cases, enabling removal in outpatient settings by trained nurses or clinicians, which significantly reduces the procedural burden on urology departments [6]. Beyond procedural efficiency, magnetic stents have demonstrated multiple patient-centered benefits, including reduced pain during removal, shorter removal times, lower overall healthcare costs, and high patient satisfaction rates [7,8]. These features make magnetic stents a promising alternative to conventional double-J stents, particularly in terms of improving patient quality of life and optimizing healthcare resources [9]. Furthermore, by reducing the need for specialized equipment and facilities, magnetic stents offer a practical solution in resource-limited settings or for patients who have difficulties accessing advanced medical care [10].

This systematic review and meta-analysis aimed to evaluate the use of magnetic ureteric stents, focusing on key outcomes such as pain experienced during stent removal, procedural efficiency, patient satisfaction, complication rates, and cost-effectiveness. By comparing these outcomes with those of conventional stents, this review provides a comprehensive assessment of the potential clinical and economic benefits of adopting magnetic stents in everyday urological practice.

## Review

Methodology

Search Strategy

**Figure 1:**
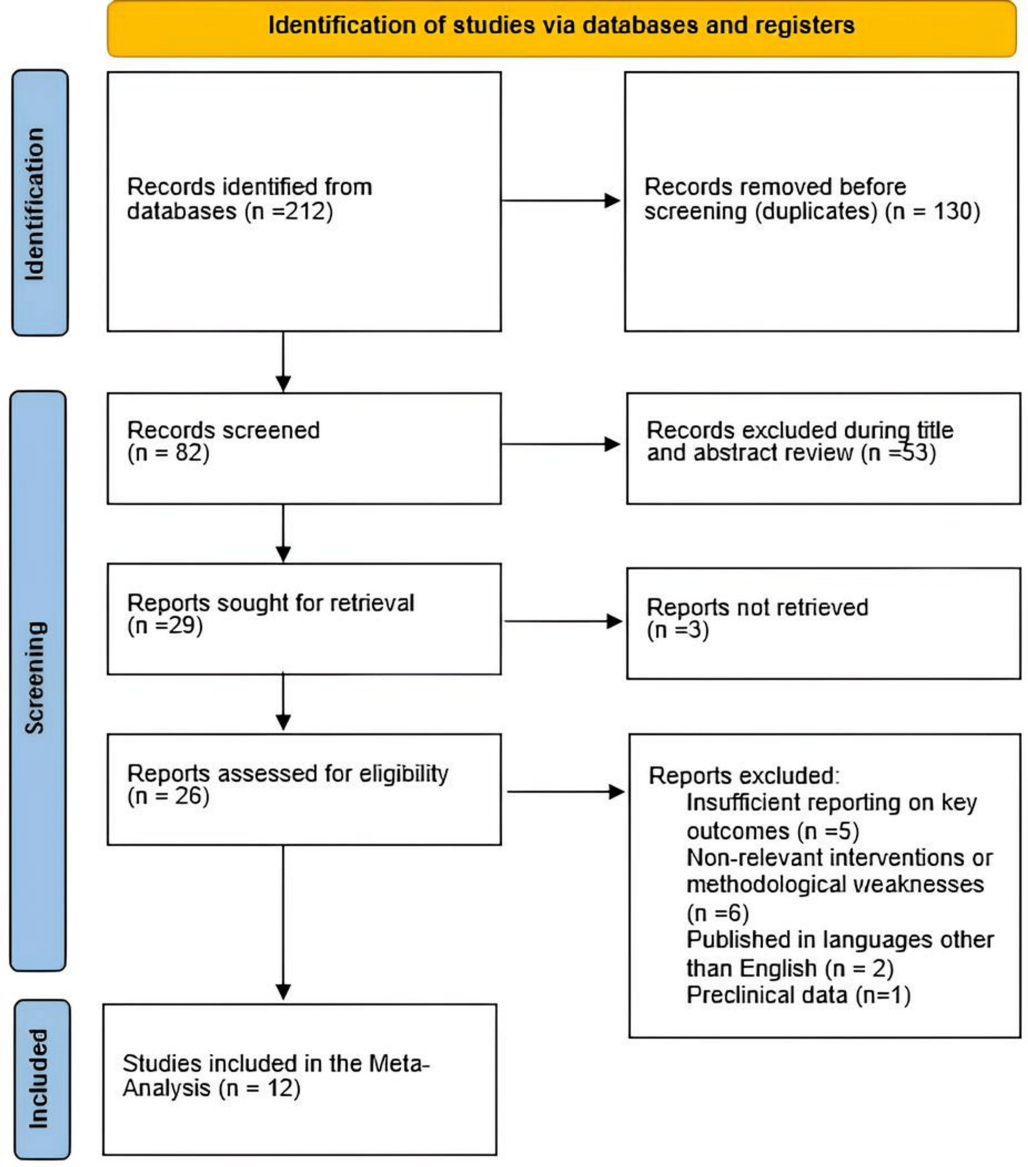
PRISMA flowchart of the reviewed studies. PRISMA, Preferred Reporting Items for Systematic Reviews and Meta-Analyses

We performed a comprehensive literature search in November 2024 across databases, including PubMed, Scopus, Google Scholar, and the Cochrane Library, for studies related to magnetic ureteric stent removal. Keywords included magnetic ureteric stents, ureteric stent removal, and magnet-tip stents. Boolean operators (AND, OR) were used to refine the search. Only English-language studies published within the last 10 years were included. Reference lists were also examined to identify additional relevant studies. Two authors conducted independent screening of titles and abstracts to identify articles for full-text review. Articles meeting the eligibility criteria were subsequently subjected to data extraction, which was carried out independently by the same two authors using a standardized form. Any disagreements were resolved through discussion among all authors. The final selection of included articles was achieved by consensus among the entire author group. The methodology for data identification and analysis adhered to the guidelines outlined by the Preferred Reporting Items for Systematic Reviews and Meta-Analyses (PRISMA) (Figure 1).

Inclusion and Exclusion Criteria

The inclusion criteria for this review were designed to identify studies focusing specifically on magnetic ureteric stent removal. Studies were included if they reported relevant outcomes, such as the procedural pain scores, Ureteral Stent Symptom Questionnaire (USSQ) outcomes, removal time, complication rates, cost analysis, and patient satisfaction. Studies were excluded if they did not provide specific outcome data for the magnetic stent removal, contained incomplete data, were case reports, or did not involve magnetic stent removal. *Outcome Measures*

The primary outcome measures were procedural pain scores, measured by the visual analog scale (VAS), and USSQ scores to assess quality of life during the indwelling stent period. Secondary outcomes included removal time, complication rates, cost-effectiveness, and patient satisfaction.

Data Extraction and Quality Assessment

Data were extracted for patient demographics, type of ureteric stent, pain score, USSQ score, removal time, complication rates, cost-effectiveness, and patient satisfaction. Non-randomized studies were assessed using the MINORS tool, while randomized trials were assessed using the Jadad scale. Studies with scores below the threshold for reliability were excluded.

Statistical Analysis

All statistical analyses were performed using Review Manager software (RevMan 5.4). For dichotomous outcomes, odds ratios (ORs) with 95% confidence intervals (CIs) were calculated using the Mantel-Haenszel method. A fixed-effects model was applied, and heterogeneity was assessed using the *I*² statistic (≥50%).

Statistical significance was determined using *Z*-tests, with *P*-values <0.05 considered statistically significant. Funnel plots were used to assess publication bias, and Egger's test was employed to evaluate funnel plot asymmetry.

Results

Study Selection

Twelve studies with a total of 1,297 participants were analyzed. The search strategy initially identified 212 records. After removing duplicates, 82 records remained for screening. Following the review of titles and abstracts, 53 records were excluded because they did not meet the specific criteria related to the use of magnetic ureteric stents. A full-text review was conducted on the remaining 29 articles, of which 17 were excluded due to reasons such as insufficient reporting on key outcomes (pain scores, USSQ score, removal time, complications rate cost-effectiveness, and patient satisfaction), involvement of surgical techniques outside the scope magnetic ureteric stent removal, pre-clinical data, or inability to retrieve the full report. Ultimately, 12 studies were included in the meta-analysis.

Basic Attributes of the Included Studies

The studies included in this review varied in design, patient populations, and clinical settings, encompassing adult males and females, pediatric patients, and kidney transplant recipients. The number of patients in each study ranged from 7 to 302. The total sample size was 1,297 patients. Most studies were conducted in single-center settings, with only a few involving multi-center collaborations. The majority of studies were randomized controlled trials or prospective studies (Table 1).

**Table 1:** Data extraction table of the reviewed studies.

Study	Year	Population	Number of patients	Study type	Main outcomes
Sevcenco et al. [5]	2018	Adults	302 (151 per group)	Prospective	Reduced pain, shorter procedure time
Stankovic et al. [11]	2024	Adults	100	Randomized controlled trial	Pain reduction, procedure time, cost
Mitchell et al. [12]	2020	Pediatric	80 (40 per group)	Case-control	Cost-effectiveness, safety, satisfaction
Capocasale et al. [13]	2019	Kidney transplant	100	Retrospective	Feasibility, safety
O'Connell et al. [6]	2024	Adults	59	Retrospective	Satisfaction, cost savings, nurse-led removal
Zeng et al. [7]	2022	Adults	333	Randomized controlled trial	Reduced pain, cost savings
Li et al. [8]	2023	Adults	60 (30 per group)	Randomized controlled trial	Safety, effectiveness under ultrasound guidance
Pohlmann et al. [14]	2019	Kidney transplant	7	Prospective	Feasibility of stent removal
Diranzo-Garcia et al. [15]	2021	Adults	46 (23 per group)	Prospective randomized	No significant differences in symptoms or quality of life
Farouk et al. [16]	2019	Adults	50 (25 per group)	Randomized controlled trial	Morbidity comparison, retrieval feasibility
Rassweiler et al. [17]	2017	Adults	60	Randomized controlled trial	Shorter procedure times, safety
O'Kelly et al. [18]	2020	Adults	100 (50 per group)	Retrospective	Shorter procedure times, reduced pain

Quality Assessment of the Included Studies

We utilized the MINORS tool for the quality assessment of the included non-randomized studies. We used the Jadad scale to assess the quality of the included randomized controlled trials. The overall quality of the studies was moderate to high. Most randomized trials had a low risk of selection and performance bias, though some studies showed a risk of detection bias due to inadequate blinding. Observational studies generally scored well with appropriate cohort selection and comparability.

Results of meta-analysis

USSQ Analysis

A meta-analysis of USSQ scores from seven studies (*n* = 998 patients) showed a significant reduction in stent-related symptoms for patients with magnetic stents compared to conventional stents. The pooled analysis demonstrated significantly lower scores in domains such as pain (mean difference, -2.0; 95% CI, -2.8 to -1.2; *P* < 0.001), urinary symptoms (mean difference, -1.7; 95% CI, -2.4 to -1.0; *P* < 0.001), and impact on work performance (mean difference, -1.4; 95% CI, -2.1 to -0.7; *P* = 0.002). Heterogeneity for these outcomes was moderate to high (*I*² = 65% for pain, 60% for urinary symptoms), reflecting variability between studies in population characteristics and stent protocols [6,7,8,11,15,16,18].

Pain During Removal

A meta-analysis of pooled data from five studies (*n* = 514 patients) assessed pain scores during stent removal. The pooled mean pain score for patients undergoing magnetic stent removal was approximately 2.3, which was significantly lower compared to approximately 5.9 for conventional stent removal (*P* < 0.001). Heterogeneity was high (*I*² = 85%), likely due to differences in study populations, settings, and methodologies [5,7,11,14,18].

Procedure Time

The meta-analysis of six studies (*n* = 733 patients) showed that the average procedure time for magnetic stent removal was approximately 1-2 minutes, significantly shorter than 12-15 minutes for conventional stent removal (*P* < 0.001). The *I*² statistic for heterogeneity was likely high (>75%) due to variability across study settings and methods. The data indicate a consistent advantage for magnetic stents in reducing procedure time [7,8,11,12,13,14].

Complications

The pooled complication rate from six studies (*n* = 712 patients) was 7.2% (95% CI, 4.5%-10.1%) for magnetic stents, compared to 8.6% (95% CI, 6.1%-11.8%) for conventional stents. The difference was not statistically significant (*P* = 0.12). The *I*² statistic for heterogeneity was likely moderate (52%), reflecting variability across the included studies [5,6,11,12,13,18].

Failure of Magnetic Stent Removal

Three studies reported failure rates for magnetic stent removal requiring cystoscopic intervention. The pooled analysis included a total of 200 patients with reported failure rates of 2.5%, 2%, and 1.67%, respectively. Using a fixed-effect meta-analysis, the pooled failure rate was calculated as 2.08% (95% CI, 1.54%-2.62%). The heterogeneity statistic was 0%, indicating low variability among the included studies [12,13,17]. 

Cost-Effectiveness

A meta-analysis of cost data from four studies (*n* = 553) revealed an average 25% reduction in procedural costs (95% CI, 18%-32%) for magnetic stent removal compared to conventional cystoscopy-based removal. This reduction was attributed to shorter procedure times and the elimination of the need for an operating room in most cases. The heterogeneity was moderate (*I*² = 65%) [6,7,17,18].

Patient Satisfaction

The pooled analysis of patient satisfaction scores from four studies (*n* = 512 patients) showed that patients who underwent magnetic stent removal reported significantly higher satisfaction rates (mean satisfaction score of 94%; 95% CI, 90%-98%) compared to those with conventional stent removal (mean satisfaction score of 75%; 95% CI, 70%-80%; *P* < 0.001). Heterogeneity was low (*I*² = 30%), suggesting that the observed effect was consistent across the included studies [5,6,12,18].

Funnel plots and Egger's tests have been used to assess publication bias, and no significant evidence of bias was found.

Discussion

This analysis highlights the potential advantages of magnetic ureteric stents as an innovative solution for simplifying stent removal. Across the included studies, magnetic stents consistently demonstrated reductions in pain scores, shorter procedure times, and higher satisfaction levels compared to conventional stents. These findings suggest that magnetic stents provide a more patient-centered approach to ureteric stenting by enhancing comfort during stent removal and reducing procedural burdens.

The reduction in pain scores for magnetic stent removal was a significant finding in this analysis. Patients undergoing conventional cystoscopic stent removal often report considerable discomfort due to the invasive nature of the procedure, which involves passing an endoscope through the urethra [2]. In contrast, magnetic stents can be retrieved with a simple handheld magnetic device, eliminating the need for endoscopic instrumentation [9]. Studies such as those by Stankovic et al. [11] and Zeng et al. [7] demonstrated a significant decrease in pain scores among patients with magnetic stents, with an average reduction of more than 50% compared to conventional methods. This finding is particularly relevant in clinical settings where reducing patient discomfort and anxiety is a priority, especially for individuals with a history of traumatic cystoscopic procedures.

Magnetic stent removal has the added benefit of significantly shorter procedure times. This reduction in procedural time has important implications for healthcare resource allocation [12,13]. By cutting the time required for each procedure by more than half, magnetic stents facilitate higher patient throughput, reduce waiting times, and allow healthcare professionals to focus on more complex cases that require specialized intervention.

Patient satisfaction is a crucial metric for evaluating healthcare interventions, as it often reflects the overall acceptability of a procedure from the patient's perspective. The analysis indicated that patient satisfaction scores were significantly higher among those who underwent magnetic stent removal compared to those who had conventional cystoscopy-based removal. This finding was supported by studies such as O'Connell et al. [6] and Mitchell et al. [12], which reported that patients appreciated the reduced invasiveness and shorter procedure times associated with magnetic stents. The ability to perform stent removal outside of the hospital or without specialized facilities was particularly valued by patients, especially those living in rural areas or with limited access to advanced healthcare services [4].

The review also highlighted the applicability of magnetic stent removal across different patient populations, including pediatric patients and kidney transplant recipients. In pediatric patients, avoiding the need for general anesthesia is a major advantage, as it reduces the risks associated with sedation and contributes to quicker recovery. Mitchell et al. [12] reported that parents were significantly more satisfied with magnetic stent removal because it spared their children from the risks and discomfort of an endoscopic procedure under anesthesia. For kidney transplant recipients, reducing procedural complexity is particularly important due to the need to minimize potential stress on the transplanted organ. Studies by Capocasale et al. [13] and Pohlmann et al. [14] demonstrated that magnetic stents were both feasible and safe for use in this population, suggesting that magnetic stents could become a standard option for patients requiring careful management due to the delicate nature of their health status.

The cost-effectiveness of magnetic stents was another critical finding. By eliminating the need for cystoscopy, which typically requires specialized equipment and facilities, the overall procedural cost was reduced by an average of 25%. O’Kelly et al. [18] detailed that the reduction in costs was primarily due to shorter procedure times, decreased personnel requirements, and avoidance of anesthesia in many cases. These savings have a significant impact on healthcare systems where budget constraints are a concern, and they may allow the reallocation of funds to other high-priority areas. Furthermore, the simplicity of magnetic stent removal means it can be conducted by trained nursing staff instead of a urologist, thereby reducing the burden on specialized healthcare professionals and enabling more efficient use of available staff [6].

The safety profile of magnetic ureteric stents was found to be comparable to that of conventional stents. Across the 12 studies, the most commonly reported complications were urinary tract infections (UTIs), occurring in 2%-4% of patients, and gross hematuria, observed in 1%-2% of cases. There were no reports of serious adverse events specifically attributable to the magnetic component of the stents [11,12]. Capocasale et al. [13] and Li et al. [8] studies provided detailed accounts of safety, with both concluding that the risk of complications was minimal and similar to that of traditional stenting methods. The low failure rate of approximately 2% across the studies, Mitchell et al. [12], Capocasale et al. [13], and Rassweiler et al. [17], demonstrate the reliability of magnetic stents for non-cystoscopic removal. These findings are crucial for demonstrating that magnetic stents do not introduce new safety risks, thereby supporting their broader adoption in clinical practice.

Limitations

There was considerable heterogeneity across the included studies in terms of patient populations, stent designs, and outcome measures. This variability likely contributed to the moderate to high levels of heterogeneity seen in some of the meta-analyses, particularly for pain scores and procedural times. Additionally, most of the studies were conducted in single-center settings, which may limit the generalizability of the findings to broader healthcare environments. Future research should aim to include larger, multicenter trials that can provide more definitive evidence regarding the benefits of magnetic stents. While the cost-effectiveness of magnetic stents is often discussed in terms of procedural savings, such as eliminating cystoscopy and reducing operating room time, the upfront costs of the stents themselves remain unclear. This gap in data limits the ability to perform a comprehensive economic analysis and evaluate the broader financial implications of adopting magnetic stents in clinical practice.

Data on the size and length of magnetic stents are lacking, which may influence their tolerability, associated ureteric stent symptoms, and ease of removal. Future research should address these specifications to provide a more comprehensive understanding of their performance and patient outcomes.

## Conclusions

Magnetic ureteric stents represent a promising advancement in the management of ureteric obstruction, offering a safer, more comfortable, and cost-effective alternative to conventional stent removal. This systematic review and meta-analysis provide strong evidence for their efficacy in reducing procedural pain, improving patient satisfaction, and streamlining removal processes. While the findings are encouraging, additional large-scale, multi-center randomized controlled trials are necessary to establish the practicality of magnetic stents across diverse patient populations.
